# Supplementary material for: Metabolomics Benefits from Orbitrap GC–MS—Comparison of Low- and High-Resolution GC–MS
Source: Metabolites. 2020 Apr 4;10(4):143. doi: 10.3390/metabo10040143 (PMC7254393; doi:10.3390/metabo10040143)
Supplement: Supplementary file 1 [file metabolites-10-00143-s001.zip › metabolites-681227.pdf]

metabolites

Supplementary

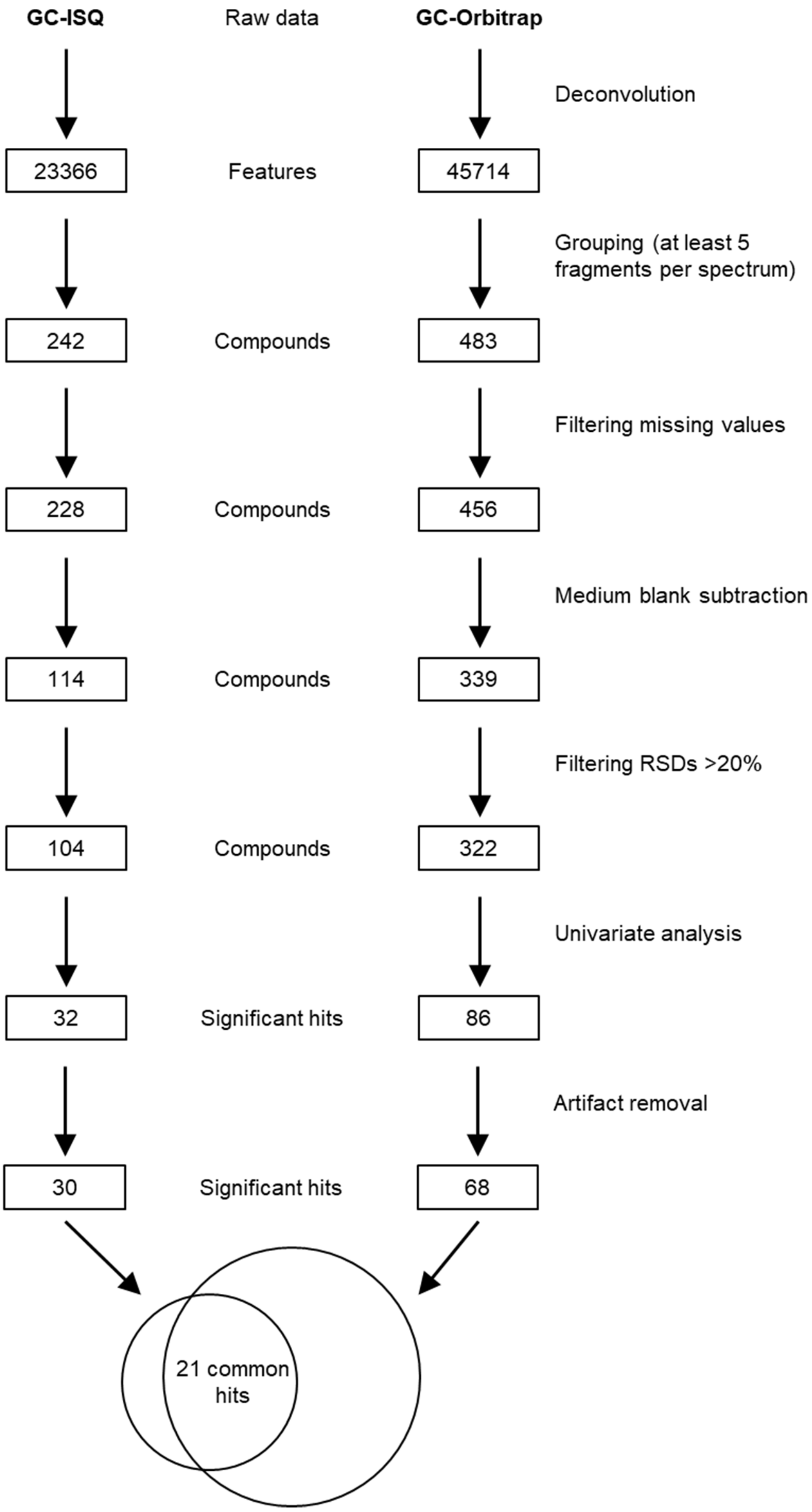

Figure S1: Effect of data curation steps of feature/compound number. Raw data was deconvoluted using XCMS. Grouping *m*/*z* features to compounds was done using CAMERA, metaMS and an in-lab script. Filtering missing values was done within the workflow of statTarget. RSD filtering and medium blank subtraction was done manually in MS Excel. Univariate analysis was carried out using Metaboanalyst. Artifacts were removed from significant hits after manually checking raw data.

Table S1. Evaluation of reproducibility of peak areas on both systems. Relative standard deviations (RSD) of peak areas of 13 fatty acid methyl esters (FAMEs) at retention times (RT) throughout the 40 min method were calculated. Both systems were flushed with the same sample four times prior to measurements of four technical replicates.

	GC-ISQ		GC-Orbitrap	
	RT	RSD (%)	RT	RSD (%)
FAME01	8.34	1.4	8.61	2.0
FAME02	10.30	1.1	10.63	3.2
FAME03	12.41	1.4	12.77	2.9
FAME04	14.57	2.1	14.96	2.9
FAME05	16.71	2.9	17.11	3.1
FAME06	18.78	4.6	19.22	3.4
FAME07	20.78	6.9	21.24	3.3
FAME08	22.72	10.2	23.19	3.9
FAME09	24.57	10.7	25.06	3.7
FAME10	28.07	11.8	28.58	5.2
FAME11	29.71	14.3	30.24	5.3
FAME12	31.29	14.4	31.79	4.3
FAME13	33.75	20.3	34.18	4.4

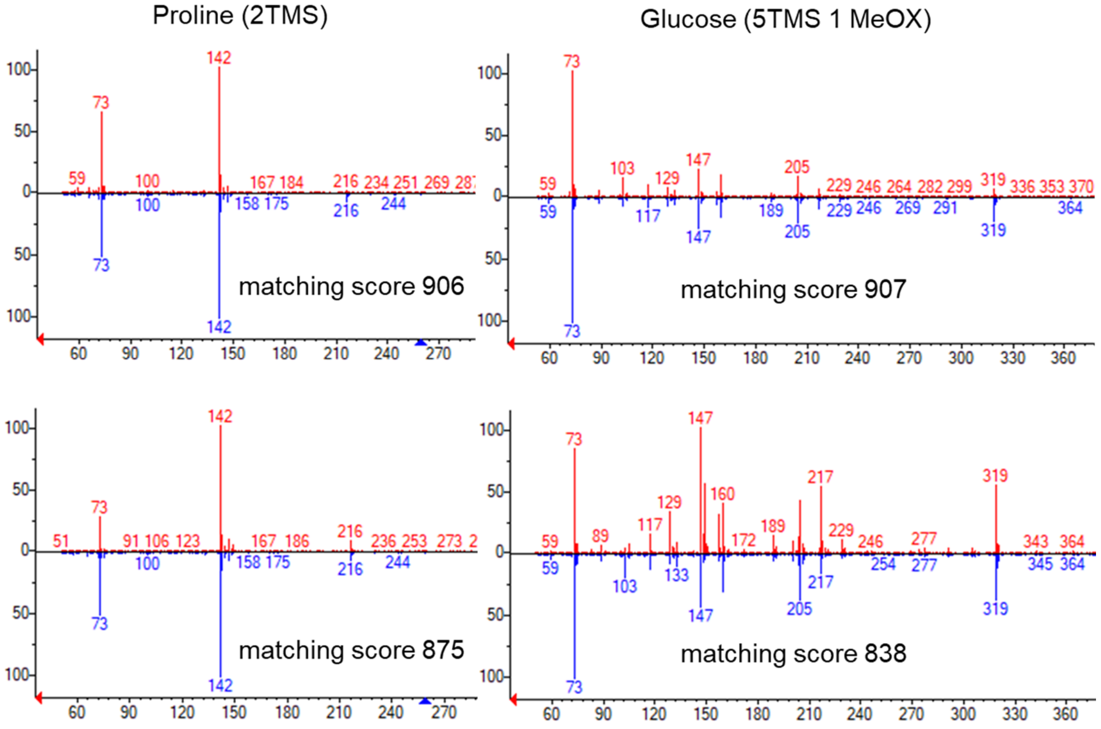

Figure S2. Comparison of mass spectra between Orbitrap and ISQ. High quality mass spectra of proline and glucose taken from raw data from the ISQ GC-MS (top) and the Orbitrap GC-MS (bottom) give different matching scores when comparing with spectral databases due to different relative intensities of fragment ions.

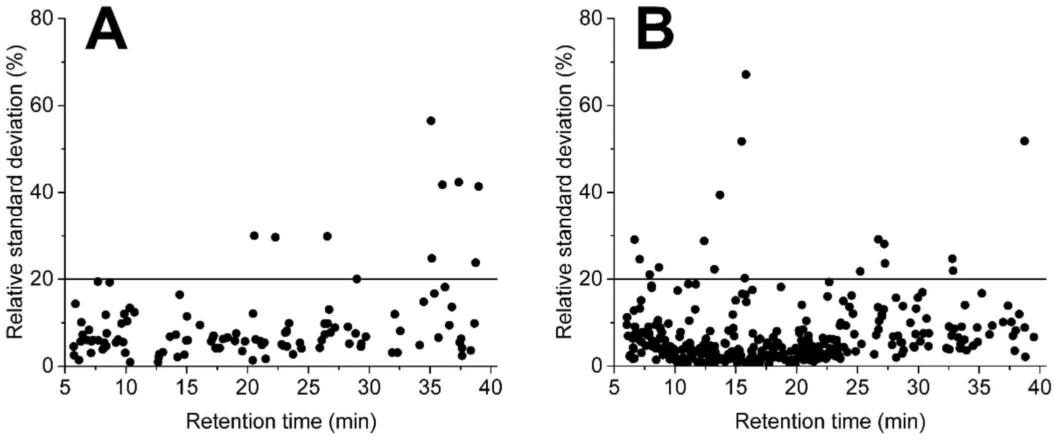

Figure S3: Comparison of the analytical variability in the comparative metabolomics experiment between the ISQ GC-MS (A) and the Orbitrap GC-MS (B). Each dot represents a compound eluting at a certain retention time. RSDs were calculated from repeat injection of pooled QC samples throughout the measurement sequence. Shown here are remaining compounds after missing value removal and blank subtraction. Compounds exceeding the threshold of 20% RSD were excluded from further analysis.

© 2020 by the authors. Submitted for possible open access publication under the terms and conditions of the Creative Commons Attribution (CC BY) license (http://creativecommons.org/licenses/by/4.0/).
